# Clinical characteristics of SARS-CoV-2 pneumonia diagnosed in a primary care practice in Madrid (Spain)

**DOI:** 10.1186/s12875-021-01430-y

**Published:** 2021-04-29

**Authors:** Marina Guisado-Clavero, Ana Herrero Gil, Marta Pérez Álvarez, Marta Castelo Jurado, Ana Herrera Marinas, Vanesa Aguilar Ruiz, Ileana Gefaell Iarrondo, Miguel Menéndez Orenga, Sara Ares-Blanco

**Affiliations:** 1 Federica Montseny Primary Care Centre. Av. Albufera 285, 28035 Madrid, Spain; 2 Accident and Emergency Department, Hospital Infanta Leonor, Av. Gran Vía del Este, 80, 28031 Madrid, Spain; 3 Research Institute I+12 (CIBERESP), 12 de Octubre Hospital, Av. de Córdoba, s/n, 28041 Madrid, Spain

**Keywords:** COVID-19, SARS-CoV-2, Pneumonia, Primary Care

## Abstract

**Background:**

Possible cases of SARS-CoV-2 infection were diagnosed in primary care in Madrid, some of these cases had pneumonia. Most of the SARS-CoV-2 pneumonia published data came from hospitalised patients. This study set out to describe clinical characteristics of patients with SARS-CoV-2 pneumonia diagnosed in primary care across age groups and type of pneumonia.

**Methods:**

Observational retrospective study obtaining clinical data from the electronic health records of patients who were followed-up by SARS-CoV-2 possible infection in a primary care practice in Madrid. All the cases were collected by in-person or remote consultation during the 10th March to the 7th of April. Exposure: Diagnosis of SARS-CoV-2 pneumonia by chest X-ray ordered by the GP. Main outcomes and measures: Symptoms of SARS-CoV-2 pneumonia, physical examination and diagnostic tests as a blood test, nasopharyngeal swab results for RT-PCR (Reverse transcriptase-polymerase chain reaction) and chest X-ray results.

**Results:**

The overall SARS-CoV-2 pneumonias collected were 172 (female 87 [50.6%], mean age 60.5 years standard deviation [SD] 17.0). Comorbidities were body mass index ≥ 25 kg/m^2^ (90 [52.3%]), hypertension (83 [48.3%]), dyslipidaemia (68 [39.5%]) and diabetes (33 [19.2%]). The sample was stratified by age groups (< 50 years, 50–75 years and ≥ 75 years). Clinical manifestations at onset were fever (144 [83.7%]), cough (140 [81.4%]), dyspnoea (103 [59.9%]) and gastrointestinal disturbances (72 [41.9%]). Day 7.8 (SD:4.1) from clinical onset was the mean day of pneumonia diagnosis. Bilateral pneumonia was more prevalent than unilateral (126 [73.3%] and 46 [26.7%]). Patients with unilateral pneumonia were prone to higher pulse oximetry (96% vs 94%, *p* < 0.001). We found differences between unilateral and bilateral cases in C-reactive protein (29.6 vs 81.5 mg/L, *p* < 0.001), and lymphocytes (1400.0 vs 1000.0E3/ml, *p* < 0.001). Complications were registered: 42 (100%) of patients ≥ 75 years were admitted into hospital; pulmonary embolism was only present at bilateral pneumonia (7 patients [5.6%]) and death occurred in 1 patient with unilateral pneumonia (2.2%) vs 10 patients (7.9%) with bilateral pneumonia ( *p* 0.170).

**Conclusion:**

Clinical manifestations of SARS-CoV-2 pneumonia were fever, cough and dyspnoea; this was especially clear in the elderly. We described different characteristics between unilateral and bilateral pneumonia.

**Supplementary Information:**

The online version contains supplementary material available at 10.1186/s12875-021-01430-y.

## Background

The infection by the novel coronavirus SARS-CoV-2 is known as COVID-19 [1]. The World Health Organization declared the SARS-CoV-2 infection a pandemic on March 11^th^, 2020. Two days later, the Spanish government announced community transmission in the country and declared a national lockdown [2–4].

The incubation period of SARS-CoV-2 can vary from 2 to 14 days [5]. Almost 80% of infected people had mild symptoms or were asymptomatic. and 20% presented with severe symptoms which required hospital assistance [6]. Mild SARS-CoV-2 disease did not require hospital admissions, but these cases could transmit the virus [7]. The most commonly reported symptoms were fever, dry cough and dyspnoea, but it could be suspected if a patient referred digestive symptoms as diarrhoea or nausea, cutaneous exanthema or even neurosensory symptoms as headache, anosmia or ageusia [5, 8, 9]. Severe symptoms developed between day 7 and day 9 from the clinical onset with typical symptoms like fever, dyspnoea and pneumonia in the chest X-ray [9].

Spain reported 250,273 total cases till the 21^st^ of May, confirmed by detection of viral RNA via reverse transcription-polymerase chain reaction (RT-PCR), of which 53.8% developed SARS-

CoV-2 pneumonia and 6.8% acute respiratory distress syndrome [10].

Other SARS-CoV-2 infection features were the dissociation between physical examination and chest X-ray findings described in hospital data [11]. Also, the most common laboratory characteristics in SARS-CoV-2 cases were high C-reactive protein (CRP) (58.3%, 95% confidence interval (CI) 21.8–94.7%), lymphopenia (43.1%, 95%CI 18.9–67.3) [12], increased fibrinogen and D-dimer [9, 13]. These last parameters had been suggested as risk factors for worst outcomes in SARS-CoV-2 infection; related to thromboembolism [14, 15]. Finally, some comorbidities had been reported as risk factors for poor prognosis like hypertension, diabetes, obesity, cardiovascular and respiratory chronic diseases [13, 16, 17].

In the European Surveillance System, Spain has been on the top three of most COVID-19 official cases reported [18]. Variations throughout countries could be related to differences in health care systems. Strong primary health care not only has been associated with better health outcomes in chronic diseases [19] but with offering patient-centred care [20]. In Spain, primary care has been key to detect presymptomatic and symptomatic cases. In the Madrid region, early detection of close contacts from infected cases and mild cases were followed by their primary care practice (PCP) from the outbreak onset. Despite the role of PCP to control the SARS-CoV-2 outbreak in the community, the literature about outpatient SARS-CoV-2 cases is scarce in primary care. As GPs we wanted to know when SARS-CoV-2 pneumonia appeared and which symptoms and signs could help us to identify it earlier. Thus, the study aimed to describe the characteristics of patients who were diagnosed of pneumonia associated with SARS-CoV-2 in a PCP in Madrid (Spain) and to compare differences across the ages as well as between unilateral versus bilateral pneumonia presentations.

## Methods

### Study design

**Fig. 1:**
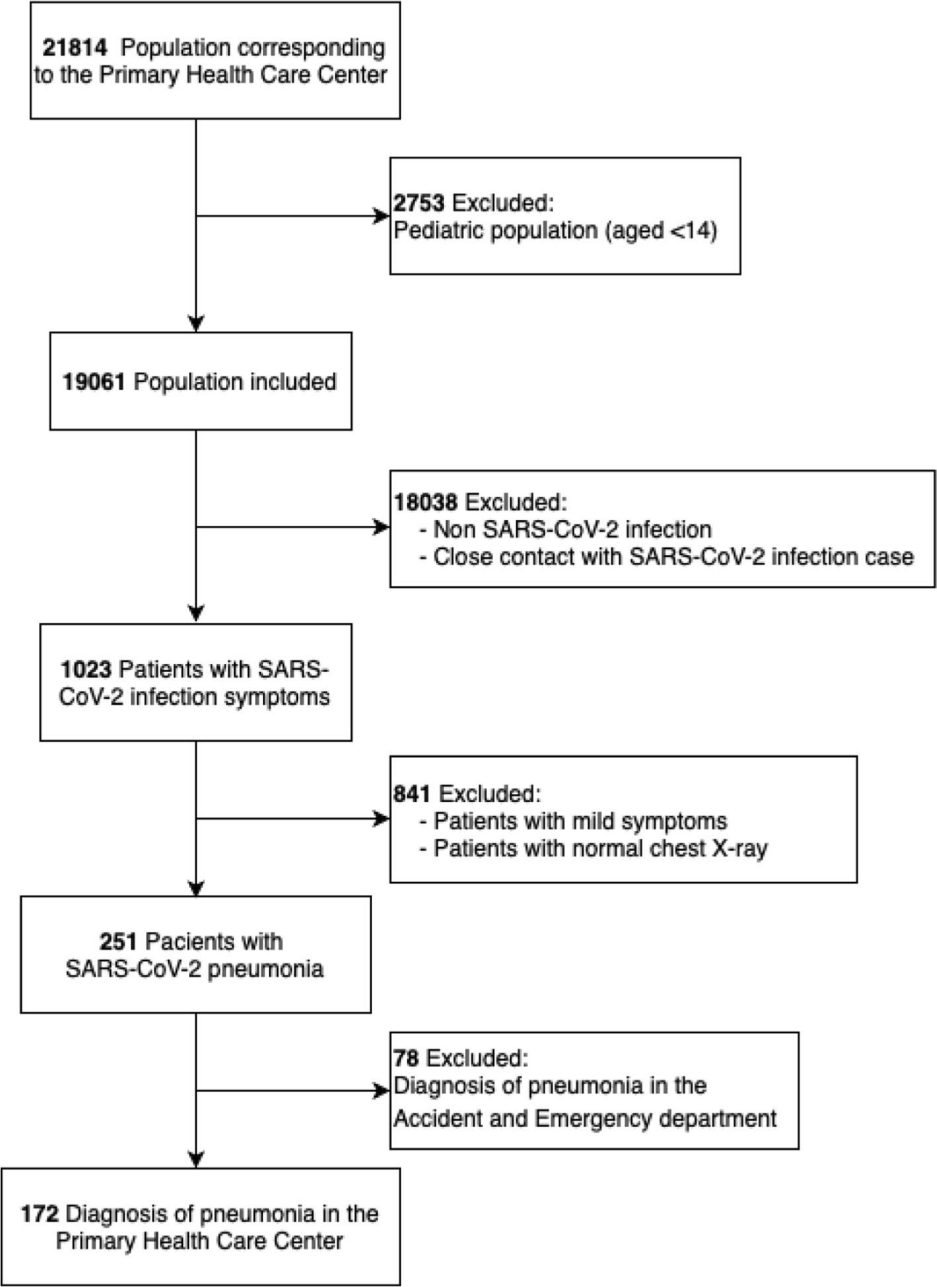
Flow chart

A retrospective observational study was conducted at Federica Montseny PCP at Madrid. This PCP is responsible to attend 21,814 people, 172 patients were included in this study (Fig. 1). The information was obtained from the electronic health record (EHR). The pneumonia cases due to SARS-CoV-2 infection were collected from those patients classified as possible cases who were examined by the PCP from the 10^th^ of March to the 7^th^ of Abril of 2020. Patients were eligible for the study if they were over 14 years old and the SARS-CoV-2 pneumonia was diagnosed by their general practitioner ( GP) at PCP. We excluded patients whose diagnosis was made directly at the hospital without PCP intervention and those who did not belong to Federica Montseny PCP.

### SARS-CoV-2 protocol in Madrid region

Patients were encouraged to phone their PCP and stay at home until their GP phoned them on the same day. In mild cases, patients received followed-up appointments at home by remote assessment using daily phone calls. In the case their symptoms worsened, they were advised to come to the PCP for examination where GPs would recommend taking a chest X-ray to rule out pneumonia. The criteria for requiring a chest X-ray were any COVID-19 progression symptom: persistent cough, thoracic pain, dyspnoea, persistent corporal temperature (T > 37.3º) or physical abnormalities (tachypnoea, pulse oximetry ≤ 93%, abnormal lung auscultation). The X-ray department was in the same building than our practice. If pneumonia was diagnosed, the patient was referred to Accident and Emergency department (A&E) for laboratory tests and treatment. Mild pneumonia cases were followed-up by the PCP in conjunction with A&E team. Mild pneumonia was defined mainly as unilateral pneumonia as well as some cases of bilateral pneumonia with local patchy opacities; these patients did not have comorbidities; they were stable with good oximetry and without bad prognosis signs in their laboratory findings. The severe cases were referred straight to the A&E by their GPs. RT-PCR was not accessible at primary care so patients were referred to A&E department at the diagnosis to be tested, some of the patients were admitted in hospitals where we coud not check the RT-PCR result.

### Variables

The main result was the type of pneumonia: unilateral or bilateral defined by chest X-ray results. Other variables were sociodemographic, comorbidities, prescribed chronic drugs, clinical characteristics of SARS-CoV-2 infection and laboratory results.

### Statistical analysis

Descriptive analyses were carried out for demographic, personal background, physical examination and diagnostic tests, treatment and consequences of SARS-CoV-2 infection. Quantitative variables were expressed as means with standard deviation (SD) or by medians with interquartile range (IRQ), qualitative variables were expressed as percentages. Differences were evaluated using Chi-square test for categorical variables, T-test or ANOVA for normally distributed variables and Wilcoxon-Mann–Whitney or Kruskal–Wallis test for non-normal variables. For all the statistical analyses, *p-*value (*p)* < 0.05 was considered significant. Data were stratified by age group (< 50 years, 50–75 years, ≥ 75 years) and pneumonia unilateral or bilateral. All data were anonymised following national and international laws. All analyses were performed using STATA 16 and R 3.4.4.

## Results

The PCP diagnosed and followed-up 1,023 patients with clinical symptoms of SARS-CoV-2 infection, SARS-CoV-2 pneumonia was present in 172 of those patients (Fig. 1). As shown in Table 1, 87 (50.6%) of the patients were female. The mean age of these 172 patients was 60.5 years. The most frequent comorbidities were body mass index ≥ 25 kg/m^2^ (BMI) (90 patients [52.3%]), hypertension (83 patients [48.3%]), dyslipidaemia (68 patients [39.5%]) and type 2 diabetes (33 patients [19.2%]). As expected, comorbidities increased with the patient´s age; being those ≥ 75 years who had a higher burden of disease. The prevalence of pulmonary thromboembolism was 4.1% (7 patients) without differences across the age distribution or sex, however, death was related to age (2 patients [2%] in 50–75 years old vs 9 patients [21%] of ≥ 75 years and no deaths < 50 years, *p* < 0.001).

**Table 1:** Clinical characteristics of SARS-CoV-2 pneumonia stratified by age groups

Age groups:	All	< 50 years	50–75 years	≥ 75 years	*p* value
Total number, n (%)	172 (100)	48 (27.9)	82 (47.7)	42 (24.4)	
**Sociodemographic variables**					
Age, mean (SD), years	60.5 (17.0)	39.0 (8.3)	62.1 (6.5)	81.9 (5.5)	< 0.001
Sex, n (%)					
Female	87 (50.6)	25 (52.0)	32 (39)	30 (71)	0.003
Male	85 (49.4)	23 (48.0)	50 (61)	12 (29)	
**Comorbidities**					
Cardiovascular risk factor					
Smoke habit, n (%)	13 (7.6)	3 (6)	10 (12)	0 (0)	0.062
BMI ≥ 25 kg/m^2^, n (%)	90 (52.3)	16 (33)	51 (62)	23 (55)	0.012
Hypertension, n (%)	83 (48.3)	6 (12)	39 (48)	38 (90)	< 0.001
type 2 Diabetes, n (%)	33 (19.2)	0 (0)	21 (26)	12 (29)	< 0.001
Dyslipidemia, n (%)	68 (39.5)	11 (23)	32 (39)	25 (60)	0.002
Respiratory diseases					
Asthma, n (%)	21 (12.2)	4 (8)	7 (9)	10 (24)	0.031
COPD, n (%)	9 (5.2)	1 (2)	5 (6)	3 (7)	0.50
Cardiovascular diseases					
Ischaemic heart diseases, n (%)	9 (5.2)	0 (0)	3 (4)	6 (14)	0.007
Arrhythmias, n (%)	10 (5.8)	1 (2)	2 (2)	7 (17)	0.003
Heart failure, n (%)	7 (4.1)	0 (0)	3 (4)	4 (10)	0.072
Other diseases					
Chronic kidney diseases, n (%)	12 (7.0)	0 (0)	3 (4)	9 (21)	< 0.001
Cognitive impairment, n (%)	10 (5.8)	1 (2)	1 (1)	8 (19)	< 0.001
Cancer, n (%)	10 (5.8)	0 (0)	4 (5)	6 (14)	0.014
Rheumatological diseases, n (%)	14 (8.1)	3 (6)	6 (7)	5 (12)	0.58
**Chronic treatment**					
no drugs, n (%)	33 (19.2)	19 (40)	14 (17)	0 (0)	< 0.001
1–4 drugs, n (%)	68 (39.5)	25 (52)	35 (43)	8 (19)	
5–9 drugs, n (%)	43 (25.0)	3 (6)	20 (24)	20 (48)	
≥ 10 drugs, n (%)	28 (16.3)	1 (2)	13 (16)	14 (33)	
**Antithrombotic/anticoagulant treat**					
Antithrombotic drug, n (%)	14 (8.1)	1 (2)	7 (9)	6 (14)	0.019
Anticoagulant drug, n (%)	8 (4.7)	1 (2)	2 (2)	5 (12)	
Non consumption, n (%)	150 (87.2)	46 (96)	73 (89)	31 (74)	
**Symptoms**					
Fever, n (%)	144 (83.7)	41 (85)	74 (90)	29 (69)	0.003
Cough, n (%)	140 (81.4)	43 (90)	68 (83)	29 (69)	0.039
Dyspnea, n (%)	103 (59.9)	29 (60)	50 (61)	24 (57)	0.91
Gastrointestinal disturbances, n(%)	72 (41.9)	23 (48)	32 (39)	17 (40)	0.60
Myalgias, n (%)	51 (29.7)	15 (31)	30 (37)	6 (14)	0.035
Thoracic Pain, n (%)	27 (15.7)	17 (35)	9 (11)	1 (2)	< 0.001
Pleural chest Pain, n (%)	10 (5.8)	4 (8)	6 (7)	0 (0)	0.17
Rhinitis, n (%)	8 (4.7)	4 (8)	4 (5)	0 (0)	0.17
Odynophagia, n (%)	20 (11.6)	8 (17)	8 (10)	4 (10)	0.44
Asthenia, n (%)	47 (27.3)	13 (27)	20 (24)	14 (33)	0.57
Headache, n (%)	47 (27.3)	13 (27)	20 (24)	14 (33)	0.57
Dysgeusia, n (%)	9 (5.2)	6 (12)	3 (4)	0 (0)	0.020
Anosmia, n (%)	2 (1.2)	1 (2)	1 (1)	0 (0)	0.65
**Symptoms categorized**					
1–3 total symptoms, n (%)	97 (56.7)	20 (42)	41 (51)	36 (86)	< 0.001
≥ 4 total symptoms, n (%)	74 (43.3)	28 (58)	40 (49)	6 (14)	
**Physical examination**					
Temperature, median (IQR), ºC	37.2 (36.7, 37.8)	37.3 (0.8)	37.4 (0.8)	37.0 (0.8)	0.060
Heart rate, mean (SD), bpm	93.2 (16.1)	99.6 (13.8)	93.3 (16.5)	85.9 (15.1)	< 0.001
Respiratory rate, (median (IQR), rpm	16.0 (15.0, 21.0)	16.0 (15.0, 16.0)	18.0 (15.5, 21.0)	22.0 (15.0, 25.0)	0.028
Pulse oximetry, median (IQR), %	94.0 (92.0, 96.5)	96.0 (94.0, 97.5)	94.0 (92.0, 96.0)	92.0 (88.5, 94.0)	< 0.001
Normal lung auscultation, n (%)	38 (22.1)	13 (27)	21 (26)	4 (10)	0.16
Abnormal lung auscultation, n (%)	54 (31.4)	12 (25)	24 (29)	18 (43)	0.16
**Blood test**					
CRP, median (IQR), mg/L	65.3 (29.0, 128.0)	57.0 (12.8, 115.0)	60.9 (34.0, 135.0)	81.5 (45.0, 133.0)	0.32
Lymphocytes, median (IQR), 10e3/ml	1100.0 (800.0, 1500.0)	1250.0 (1000.0, 1750.0)	1000.0 (800.0, 1400.0)	900.0 (600.0, 1200.0)	0.002
D-Dimer, median (IQR), μg/L	445.0 (288.0, 910.0)	410.0 (240.0, 901.5)	460.0 (270.0, 880.0)	464.0 (320.0, 2160.0)	0.39
Fibrinogen ≥ 500, n (%), mg/dL	127 (73.8)	31 (65)	62 (76)	34 (81)	0.29
Ferritin, median (IQR), μg/L	443.5 (200.0, 1215.0)	474.0 (320.0, 1738.0)	494.0 (203.0, 913.0)	325.5 (184.5, 1006.5)	0.73
**RT-PCR SARS-CoV-2**					
Negative, n (%)	36 (20.9)	17 (35)	13 (16)	6 (14)	0.041
Positive, n (%)	122 (70.9)	27 (56)	64 (78)	31 (74)	0.042
**Pneumonia features**					
Pneumonia onset, mean (SD), days	7.8 (4.1)	8.1 (4.7)	8.3 (3.8)	6.7 (3.7)	0.12
Unilateral, n (%)	46 (26.7)	21 (44)	19 (23)	6 (14)	0.003
Bilateral, n (%)	126 (73.3)	27 (56)	63 (77)	36 (86)	0.004
**Complications**					
Hospital admission, n (%)	140 (81.4)	30 (62)	68 (83)	42 (100)	< 0.001
Pulmonary thromboembolism, n (%)	7 (4.1)	2 (4)	2 (2)	3 (7)	0.45
Death, n (%)	11 (6.4)	0 (0)	2 (2)	9 (21)	< 0.001

*n* number, *SD* standard deviation, *BMI* body mass index, *COPD* chronic obstructive pulmonary disease, *IQR* interquartile range, *CRP* C-reactive protein, *RT-PCR* reverse transcription PCR, *bmp* beats per minute, *rpm* respiratory rate per minute

### Clinical manifestations

**Fig. 2:**
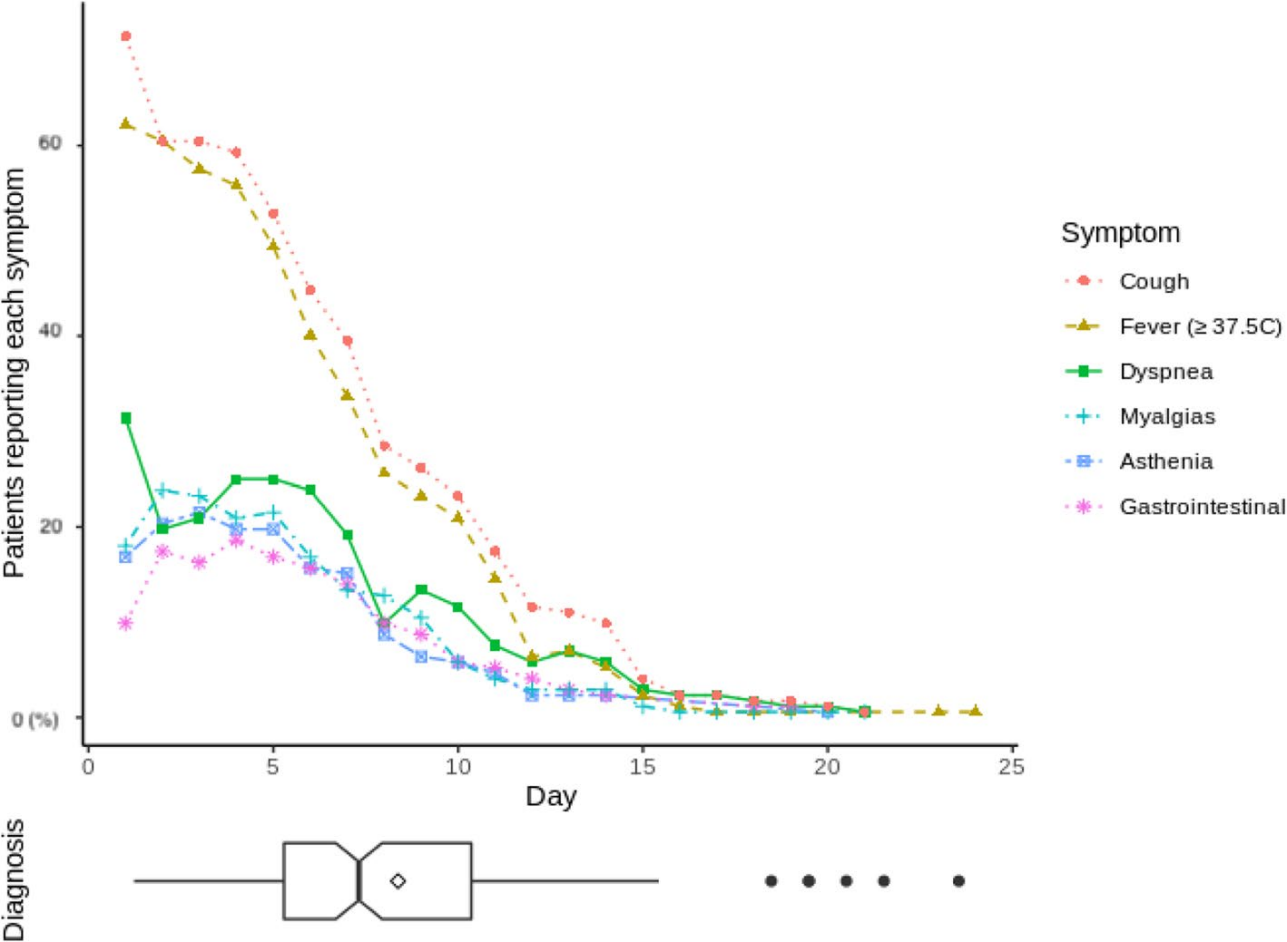
Chronological line of clinical manifestations in SARS-CoV-2 pneumonia in the whole sample (n: 172)

**Fig. 3:**
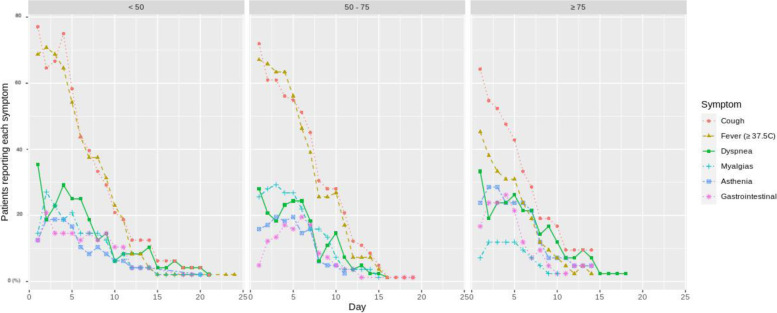
Chronological line of clinical manifestations in SARS-CoV-2 pneumonia stratified by age groups (< 50 years, 50–75 years, ≥ 75 years)

Fever (144 patients [83.7%]) and cough (140 patients [81.4%]) were the most prevalent symptoms followed by dyspnoea (103 patients [59.9%]) and gastrointestinal disturbances (72 patients [41.9%]). We did not only describe the evolution of the symptoms during the infection till the onset of pneumonia (Fig. 2) but also across the different age groups (Fig. 3); we found myalgia and asthenia also common symptoms in the evolution. A box plot of pneumonia is represented on the bottom of Fig. 2, locating the median diagnosed day in the day 7 of clinical onset and the 50% of the sample from the day 5 to day 10.

We found clinical differences in the pneumonia syndrome among the different ages; not only did ≥ 75 years have 1–3 symptoms (36 patients [86.0%]) more than < 50 years (20 patients [42.0%]) (*p* < 0.001) but their pneumonia onset was earlier, with a mean day 6.7 (SD 3.7) than the other groups (day 8.1 (SD 4.7) in < 50 years and day 8.3 (SD 3.8) in 50–75 years respectively). The group of < 50 years described ≥ 4 symptoms in 28 patients (58.0%) compared to the other groups (40 patients [49.0%] in 50–75 years and 6 patients [14.0%] in ≥ 75 years) (*p* < 0.001). The full description of the patients who did not survive can be checked in Additional file 1.

### Physical examination and laboratory tests

The physical examination was not completed in all the cases; pulse oximetry was recorded in 162 patients (94.1%), heart rate in 138 patients (80.2%), the temperature in 131 patients (76.1%), lung auscultation in 92 patients (53.4%) and respiratory rate in 60 patients (34.8%). Basal pulse oximetry had a median value of 94.0% (IQR 92.0, 96.5), the median temperature was 37.2ºC (IQR 36.7, 37.8). Statistically significant differences in pulse oximetry across the different ages and also the survivors versus no survivors were found. Patients ≥ 75 years had the lowest values (92.0%) compared to < 50 years (96.0%) and 50–75 years (94.0%, *p* < 0.001) (Table 1). We also found differences comparing non-survivors with survivors (91.0% vs 94.0%) (Additional file 1). The pulse oximetry was ≥ 94% in 98 patients (57.0%) of the whole population, that was achieved by 37 patients (77.0%) of < 50 years group, 48 patients (59.0%) of 50–75 years but only 13 patients (31.0%) of ≥ 75 years group. We did not find differences in the auscultation, respiratory rate or temperature in the different ages.

Regarding the laboratory test, we did not find differences in the values except in lymphocytes. The median value for lymphocytes was 1100.0E3/ml, (IQR 800.0, 1500.0). The values were decreasing with age from 1250E3/ml (IQR 1000.0, 1750.0) in < 50 years, 1000.0 (IQR 800.0, 1400.0) in 50–75 years to 900E3/ml (600.0, 1200.0) in the ≥ 75 years group (*p* 0.002).

Nasopharyngeal swab was obtained to confirm SARS-CoV-2 infection in 91.8% of patients (n:158) and it was positive in 70.9% of patients. A negative result was more frequent in < 50 years with 17 patients (35%) compared to ≥ 75 years group with 6 patients (14%) as well as a positive result was more common in patients ≥ 75 years group with 31 patients (74%) compared to those < 50 years with 27 patients (56%) (*p* 0.041).

### Unilateral versus bilateral pneumonia

Unilateral pneumonia was present at 46 patients (26.7% [95% CI 20.3%-33.4%]) of the cases while bilateral pneumonia was found on 126 patients (73.3% [95%CI 66.9%-79.9%]) of the sample. We observed differences between the mean age in patients with unilateral versus bilateral pneumonia (53.7 vs 63.0 years) (Table 2). Patients with bilateral pneumonia had more comorbidities, however we only found statistical significance in hypertension (16 patients [34.8%] in unilateral pneumonia vs 67 patients [53.2%] in bilateral pneumonia, *p* 0.033), type 2 diabetes (4 patients [8.7%] in unilateral pneumonia vs 29 patients [23.0%] in bilateral pneumonia, *p* 0.035) or chronic kidney disease (no patients in unilateral pneumonia vs 12 patients [9.5%] in bilateral pneumonia, *p* 0.030). Patients with unilateral pneumonia presented more frequently myalgia (17 patients [37.0%]) and chest pain (10 patients [21.7%]). Bilateral pneumonia had more dyspnoea (79 patients [62.7%]), but no significant differences were found in clinical characteristics. On the one hand, patients with unilateral pneumonia were prone to normal auscultation compared to bilateral cases (16 patients [34.8%] vs 22 patients [17.5%], *p* 0.014) and higher pulse oximetry (96% vs 94%, *p* < 0.001). We found statistical significance between unilateral and bilateral cases in CRP (29.6 vs 81.5 mg/L), lymphocytes (1400.0 vs 1000.0E3/ml) and fibrinogen ≥ 500 mg/dL (24 patients [52.2%] vs 103 [81.7%]). Unilateral pneumonia had more negative RT-PCR, 17 patients (37.0%) vs 19 patients (15.1%) of bilateral ones (*p* 0.006). 20 patients (43.5%) with unilateral pneumonia required hospital admission in comparison to 120 patients (95.2%) of bilateral pneumonia however pulmonary embolism was only present at bilateral pneumonia (7 patients [5.6%]). Death was present chiefly on bilateral cases (1 patient in unilateral cases [2.2%] vs 10 patients [7.9%], *p* 0.170) (Table 2).

**Table 2:** Differences between SARS-CoV-2 pneumonia patterns: unilateral and bilateral

Pneumonia	Unilateral	Bilateral	*p*-value
Number (%)	46 (26.7)	126 (73.3)	
Pneumonia onset, mean (SD), days	8.2 (5.1)	7.7 (3.7)	0.47
**Sociodemographic variables**			
Age, mean (SD), years	53.7 (16.6)	63.0 (16.6)	
Sex, n (%)			
Female	28 (60.9)	59 (46.8)	0.10
Male	18 (39.1)	67 (53.2)	0.10
**Comorbidities**			
Cardiovascular risk factor			
Smoke habit, n (%)	4 (8.7)	9 (7.1)	0.38
BMI ≥ 25 kg/m2, n (%)	20 (43.5)	70 (55.6)	0.25
Hypertension, n (%)	16 (34.8)	67 (53.2)	0.033
type 2 Diabetes, n (%)	4 (8.7)	29 (23.0)	0.035
Dyslipidemia, n (%)	15 (32.6)	53 (42.1)	0.26
Respiratory diseases			
Asthma, n (%)	4 (8.7)	17 (13.5)	0.40
COPD, n (%)	3 (6.5)	6 (4.8)	0.65
Cardiovascular diseases			
Ischaemic heart diseases, n (%)	1 (2.2)	8 (6.3)	0.28
Arrhythmias, n (%)	1 (2.2)	9 (7.1)	0.22
Heart failure, n (%)	2 (4.3)	5 (4.0)	0.91
Other diseases			
Chronic kidney diseases, n (%)	0 (0.0)	12 (9.5)	0.030
Cognitive impairment, n (%)	3 (6.5)	7 (5.6)	0.81
Cancer, n (%)	1 (2.2)	9 (7.1)	0.22
Rheumatological diseases, n (%)	2 (4.3)	12 (9.5)	0.27
**Chronic treatment**			
no drugs, n (%)	8 (17.4)	25 (19.8)	0.60
1–4 drugs, n (%)	21 (45.7)	47 (37.3)	
5–9 drugs, n (%)	12 (26.1)	31 (24.6)	
≥ 10 drugs, n (%)	5 (10.9)	23 (18.3)	
Antithrombotic/anticoagulant treatment			
Antithrombotic drug, n (%)	1 (2.2)	13 (10.3)	0.22
Anticoagulant drug, n (%)	2 (4.3)	6 (4.8)	
Non consumption, n (%)	43 (93.5)	107 (84.9)	
**Symptoms**			
Fever, n (%)	38 (82.6)	106 (84.1)	0.81
Cough, n (%)	37 (80.4)	103 (81.7)	0.84
Dyspnea, n (%)	24 (52.2)	79 (62.7)	0.21
Gastrointestinal disturbances, n(%)	18 (39.1)	54(42.9)	0.66
Myalgias, n (%)	17 (37.0)	34 (27.0)	0.20
Thoracic Pain, n (%)	10 (21.7)	17 (13.5)	0.19
Pleural chest Pain, n (%)	6 (13.0)	4 (3.2)	0.014
Rhinitis, n (%)	4 (8.7)	4 (3.2)	0.13
Odynophagia, n (%)	7 (15.2)	13 (10.3)	0.37
Asthenia, n (%)	12 (26.1)	35 (27.8)	0.83
Headache, n (%)	12 (26.1)	35 (27.8)	0.83
Dysgeusia, n (%)	3 (6.5)	6 (4.8)	0.65
Anosmia, n (%)	1 (2.2)	1 (0.8)	0.45
**Symptoms categorized**			
1–3 total symptoms, n (%)	25 (54.3)	72 (57.6)	0.70
≥ 4 total symptoms, n (%)	21 (45.7)	53 (42.4)	
**Physical examination**			
Temperature, mean (SD), ºC	37.2 (0.8)	37.3 (0.8)	0.86
Heart rate, mean (SD), bpm	96.2 (15.1)	92.2 (16.4)	0.20
Respiratory rate, median (IQR), rpm	16.0 (14.0, 16.0)	17.0 (15.0, 22.0)	0.073
Pulse oximetry, median (IQR), %	96.0 (94.0, 97.0)	94.0 (91.0, 96.0)	< 0.001
Normal lung auscultation, n (%)	16 (34.8)	22 (17.5)	0.014
Abnormal lung auscultation, n (%)	8 (17.4)	46 (36.5)	0.014
**Blood test**			
CRP, median (IQR), mg/L	29.6 (5.0, 81.5)	81.5 (46.2, 143.0)	< 0.001
Lymphocytes, median (IQR), 10e3/ml	1400.0 (1100.0, 1880.0)	1000.0 (800.0, 1400.0)	< 0.001
D-Dimer, median (IQR), μg/L	339.0 (197.0, 464.0)	520.0 (320.0, 1030.0)	0.035
Fibrinogen ≥ 500, n (%), mg/dL	24 (52.2)	103 (81.7)	< 0.001
Ferritin, median (IQR), μg/L	618.0 (280.5, 919.0)	424.0 (200.0, 1215.0)	0.96
**RT-PCR SARS-CoV-2**			
Negative, n (%)	17 (37.0)	19 (15.1)	0.006
Positive, n (%)	25 (54.3)	97 (77.0)	
**Complications**			
Hospital admission, n (%)	20 (43.5)	120 (95.2)	< 0.001
Pulmonary thromboembolism, n (%)	0 (0.0)	7 (5.6)	0.10
Death, n (%)	1 (2.2)	10 (7.9)	0.17

*n* number, *SD* standard deviation, *BMI* body mass index, *COPD* chronic obstructive pulmonary disease, *IQR* interquartile range, *CRP* C-reactive protein, *RT-PCR* reverse transcription PCR, *bpm* beats per minute, *rpm* respiratory rate per minute

## Discussion

In this study, females were 50.3% of the population. The age distribution was asymmetric, however non-survivors belonged mainly to the elderly. The most prevalent comorbidities were hypertension, dyslipidaemia and BMI ≥ 25Kg/m^2^. Clinical manifestations of SARS-CoV-2 pneumonia were fever and cough as the main symptoms, most of the patients had only 1–3 symptoms; this was especially clear in the elderly (≥ 75 years) whose clinical presentation was mainly with 1–3 symptoms. We described different characteristics between unilateral and bilateral pneumonia.

Some publications have been reporting age as a factor for developing severe illness due to SARS-CoV-2 infection [13], our study is consistent with this observation because elderly patients had more frequently bilateral pneumonia and also morbidity compared to younger patients.These findings are in concordance to Liu et al*.* who found more bilateral pneumonia in older patients [21]. However, elderly presented fewer symptoms than those younger than 75 years because they used to have less pneumonia symptoms [22] and also their own comorbidities could overlap some COVID-19 symptoms. The comorbidities of our population (hypertension, dyslipidemia, diabetes and BMI ≥ 25Kg/m^2^) were similar to the studies that have been published [13, 17]. These results are consistent with a meta-analysis published by Yang et al. [16], they found that patients infected by SARS-CoV-2 had hypertension (21.1%) and diabetes (9.7%) as the most prevalent comorbidities. Smoking was present in 7.5% of our population, less than others. Guan et al. [6] reported 12.6% in smokers, that difference could be explained by incomplete EHR.

In this study, we described the symptoms and their chronology up until the appearance of pneumonia. We were interested in describing clinical patterns that could help us to decide when to request a chest X-ray and how to detect pneumonia. As expected, we found fever (83.8%), cough (81.5%), dyspnea (59.5%) and myalgia (30.1%) as the most frequent symptoms, consistent with the literature [9, 12] and with Sun et al. [23] who reported fever (89.1%) and cough (72.2%) as the most frequent symptoms; the differences among their percentages and ours could be explained by the accuracy in the EHR because the record was made daily.

We collected 12 symptoms, fever, cough and dyspnea were the most frequent, consistent with the meta-analysis of Rodriguez et al. who reported fever (88.7%, 95% CI 84.5–92.9%), cough (57.6%, 95%CI 40.8–74.4%) and dyspnea (45.6%, 95% CI 10.9–80.4%). We observed that most of the patients exhibited only 1–3 symptoms (56.1%). In our study the elderly had fewer symptoms, which is consistent with Niu et al*.* [24] who described less dyspnoea and cough in patients over 80 years however in their study fever (≥ 37.3ºC) was more prevalent than in our study (75% vs 69%); maybe this could be explained by the different age ranges (in their case: 50–64, 65–79 and ≥ 80 years).

In this study, the diagnosis of pneumonia was on day 7.8 of onset of the symptoms, similar to Wang et al*.* [25] where at hospital admission had a median on day 7.0 (IQR 4.0, 8.0). These periods were longer than other publications which established the diagnosis through day 3.0 (IQR 1.0, 6.0) for non-severe SARS-CoV-2 infection but on day 5.0 (IQR 2.0, 7.0) in severe infections [6]. Our study found that the diagnosis in the elderly group was made on day 6.7 of the onset. Furthermore, this group had more deaths (21%) and they were the ones who suffered more severe disease. They had tachypnoea, 92% of pulse oximetry, 43% of abnormal auscultation as well as more abnormalities in their blood tests. Most manuscripts published did not compare the differences across age groups because they were focused on severe and not severe cases. Niu et al*.* described 90.6% of oximetry and death in 18.8 of their ≥ 80 years patients which is similar to our results.

On another note, the physical examination was not recorded in all patients. At the moment, we still do not know the predictive values of symptoms and physical examination in COVID-19, especially of the lung auscultation which involves physical touch. In Spain, we suffered a shortage of personal protective equipment (PPE) for healthcare workers (40,921 of healthcare workers were infected by SARS-CoV-2 in the country till 11^th^ of May [10] which made doctors cautious of examining the patients if they could get a diagnosis through anamnesis and chest X-ray. If we compare with other series, Guan et al*.* do not detail these physical signs in their data as well as Zhou et al*.* who just describe respiratory rate in 29% of their patients [26]. A survey [27] was conducted in Canada to explore the opinions of GPs during the SARS (2003) and H1N1 (2010) outbreaks, GPs answered that they would avoid physical examinations in patients with SARS (62%) and patients with H1N1 (18%). More studies should be conducted to observe the benefit of the physical examination in the management of COVID-19. This should come along with qualitative research to understand the perspective of the doctors.

We described two clinical patterns by chest X-ray: unilateral and bilateral pneumonia however, little is known about other differences between both types of pneumonia [12]. In this study, 73.3% of the types of pneumonia were bilateral; our results are in concordance with Shi et al. (n:416) [28] and Chen et al. [29], where around 75% of cases of pneumonia corresponded to bilateral and 25% to unilateral. Shi et al*.* as well as Guan et al*.* described bilateral pneumonia more frequently in severe cases. Unilateral pneumonia appeared more frequently in younger patients (53.7 vs 62.8 years), the onset was slightly later than bilateral pneumonia (8.9 vs 7.8 days) and most of them did not have red flags on their examination (abnormal auscultation, oximetry ≤ 94%) or blood tests ( CRP ≥ 81.5 mg/L, D-Dimer ≥ 520 μg/L, Lymphocytes ≤ 1,000 10e3/L, Fibrinogen ≥ 500 mg/dL) [6, 28]. They had higher oximetry (96% vs 94%) and more normal auscultation (34.8% vs 17.5%) without differences in other physical signs or symptoms. Unilateral pneumonia presented 37% of negative RT-PCR similar to Weissleder et al*.* who reported 30% ( range:10–40%) of false negative results [30]. Any of our patients with unilateral pneumonia had a pulmonary embolism and just one of them did not survive.

Finally, health systems have faced significant stress because of pandemic, unfortunately more pandemic waves could happen till vaccination is available. This situation has highlighted the need for a whole patient perspective to take decisions especially when patients are assessed by remote consultation. Priority should be given to primary care who have a long-term relationship with their patients because not only they can follow them but they can manage those SARS-CoV-2 pneumonia patients without red flags in settings with access to laboratory tests and chest X-ray**.** We have found that unilateral pneumonia without red flags could be monitored closely in primary care without referring patients to the hospital if they can assure follow-up tightly. We hypothesise that unilateral SARS-CoV-2 pneumonia without red flags could be managed in primary care but more research is needed to characterise these clinical patterns related to the age and unilateral vs bilateral pneumonia.

### Strengths and limitations

To our knowledge, our study is the first one to describe patients with SARS-CoV-2 pneumonia diagnosed in a PCP. Besides, we described the clinical differences between bilateral and unilateral cases of pneumonia. However, limitations of this observational study should be addressed. Firstly, this study was carried out in a unique PCP, so the results might not be wildly generalisable. The cardiovascular factors could not be updated in the EHR (especially tobacco or BMI) but the comorbidities are usually updated because we have validated the diagnosis for research with success in other studies [31]. In addition, a bias should be considered because we are located in a primary health centre which include a radiology department, so that we had facilities to diagnose SARS-CoV-2 pneumonia compared to other primary health centers of Madrid. Our study described the symptoms up through the onset of pneumonia but some of unilateral pneumonia should be addressed more thoroughly. On the one hand, we have not collected whether or not they could progress to bilateral pneumonia. On the other hand, we should approach differently those unilateral pneumonia with negative RT-PCR; we should have evidence if the RT-PCR was repeated several times. Further studies should be conducted to clarify these cases. Finally, we based our pneumonia diagnosis in the radiologist description but there could be an interpersonal variability in that description.

## Conclusions

SARS-CoV-2 pneumonia had mainly fever, cough and dyspnoea symptoms, particularly in the elderly. We found two clinical patterns: unilateral and bilateral pneumonia. Unilateral pneumonia appeared more frequently in younger patients without red flags in their physical examination or laboratory tests however bilateral pneumonia was more common in elderly patients with red flags. Primary care can manage mild pneumonia through anamnesis and proper diagnostic tests.

## Supplementary Information

**Additional file 1.** Clinical characteristics between survivors and non-survivors SARS-CoV-2 pneumonia patients.

## Data Availability

The datasets generated and/or analysed during the current study are not publicly available to protect participant privacy, but are available from the corresponding author on reasonable request.

## Abbreviations

A&E: Accident and Emergency department
BMI: Body mass index
CI: Confidence interval
CRP: C-reactive protein
EHR: Electronic health record
GP: General practictioner
IRQ: Interquartile range
*p*: *P* Value
PCP: Primary care practice
RT-PCR: Reverse transcriptase-polymerase chain reaction
SD: Standard deviation
