# Supplementary material for: Clinical characteristics of SARS-CoV-2 pneumonia diagnosed in a primary care practice in Madrid (Spain)
Source: BMC Fam Pract. 2021 Apr 29;22:83. doi: 10.1186/s12875-021-01430-y (PMC8083921; doi:10.1186/s12875-021-01430-y)
Supplement: Supplementary file 1 — Additional file 1. Clinical characteristics between survivors and non-survivors SARS-CoV-2 pneumonia patients. [file 12875_2021_1430_MOESM1_ESM.docx]

Additional file 1.- Clinical characteristics between survivors and non-survivors SARS-CoV-2 pneumonia patients

|  | **Survivors** | **Non-Survivors** | ***p* value** |
| --- | --- | --- | --- |
| Total number, n (%) | 161 (93,6) | 11 (6,4) |  |
|  |  |  |  |
| **Sociodemographic variables** |  |  |  |
| Age group, mean (SD), years |  |  |  |
| <50 years | 48 (29.8) | 0 (0.0) | <0.001 |
| 50-75 years | 80 (49.7) | 2 (18.2) |  |
| ≥75 years | 33 (20.5) | 9 (81.8) |  |
| Sex, n (%) |  |  |  |
| Female | 81 (50.3) | 6 (54.5) | 0.79 |
| Male | 80 (49.7) | 5 (45.5) | 0.79 |
|  |  |  |  |
| **Comorbidities** |  |  |  |
| Cardiovascular risk factor |  |  |  |
| Smoke habit, n (%) | 12 (7.5) | 1 (9.1) | 0.44 |
| BMI ≥25 Kg/m2, n (%) | 85 (52.8) | 5 (45.5) | 0.19 |
| Hypertension, n (%) | 73 (45.3) | 10 (90.9) | 0.003 |
| type 2 Diabetes, n (%) | 30 (18.6) | 3 (27.3) | 0.48 |
| Dyslipidaemia, n (%) | 62 (38.5) | 6 (54.5) | 0.29 |
| Respiratory diseases |  |  |  |
| Asthma, n (%) | 21 (13.0) | 0 (0.0) | 0.20 |
| COPD, n (%) | 8 (5.0) | 1 (9.1) | 0.55 |
| Cardiovascular diseases |  |  |  |
| Ischaemic heart diseases, n (%) | 7 (4.3) | 2 (18.2) | 0.046 |
| Arrhythmia, n (%) | 8 (5.0) | 2 (18.2) | 0.070 |
| Heart failure, n (%) | 6 (3.7) | 1 (9.1) | 0.38 |
| Other diseases |  |  |  |
| Chronic kidney diseases, n (%) | 11 (6.8) | 1 (9.1) | 0.78 |
| Cognitive impairment, n (%) | 8 (5.0) | 2 (18.2) | 0.070 |
| Cancer, n (%) | 10 (6.2) | 0 (0.0) | 0.39 |
| Rheumatological diseases, n (%) | 13 (8.1) | 1 (9.1) | 0.91 |
|  |  |  |  |
| **Chronic treatment** |  |  |  |
| No drugs, n (%) | 33 (20.5) | 0 (0.0) | 0.018 |
| 1-4 drugs, n (%) | 65 (40.4) | 3 (27.3) |  |
| 5-9 drugs, n (%) | 36 (22.4) | 7 (63.6) |  |
| ≥ 10 drugs, n (%) | 27 (16.8) | 1 (9.1) |  |
|  |  |  |  |
| **Antithrombotic/anticoagulant treat.** |  |  |  |
| Antithrombotic drug, n (%) | 13 (8.1) | 1 (9.1) | 0.085 |
| Anticoagulant drug, n (%) | 6 (3.7) | 2 (18.2) |  |
| Non consumption, n (%) | 142 (88.2) | 8 (72.7) |  |
|  |  |  |  |
|  |  |  |  |
| **Symptoms** |  |  |  |
| Fever, n (%) | 136 (84.5) | 8 (72.7) | 0.31 |
| Cough, n (%) | 133 (82.6) | 7 (63.6) | 0.12 |
| Dyspnoea, n (%) | 94 (58.4) | 9 (81.8) | 0.12 |
| Gastrointestinal disturbances, n (%) | 79 (43.5) | 2 (18.2) | 0.10 |
| Myalgias, n (%) | 49 (30.4) | 2 (18.2) | 0.39 |
| Thoracic Pain, n (%) | 27 (16.8) | 0 (0.0) | 0.14 |
| Pleural chest Pain, n (%) | 10 (6.2) | 0 (0.0) | 0.39 |
| Rhinitis, n (%) | 8 (5.0) | 0 (0.0) | 0.45 |
| Odynophagia, n (%) | 18 (11.2) | 2 (18.2) | 0.48 |
| Asthenia, n (%) | 43 (26.7) | 4 (36.4) | 0.49 |
| Headache, n (%) | 43 (26.7) | 4 (36.4) | 0.49 |
| Dysgeusia, n (%) | 9 (5.6) | 0 (0.0) | 0.42 |
| Anosmia, n (%) | 2 (1.2) | 0 (0.0) | 0.71 |
|  |  |  |  |
| **Symptoms categorized** |  |  |  |
| 1-3 total symptoms, n (%) | 88 (55.0) | 9 (81.8) | 0.082 |
| ≥ 4 total symptoms, n (%) | 72 (45.0) | 2 (18.2) |  |
|  |  |  |  |
| **Physical examination** |  |  |  |
| Temperature, median (IQR), ºC | 37.2 (0.8) | 37.3 (0.8) | 0.97 |
| Heart rate, mean (SD), bpm | 94.0 (16.0) | 82.3 (14.5) | 0.026 |
| Respiratory rate, median (IQR), rpm | 16.0 (15.0, 19.5) | 22.0 (17.0, 24.5) | 0.31 |
| pulse oximetry, median (IQR), % | 94.0 (92.0, 96.5) | 91.0 (88.5, 92.0) | 0.004 |
| Normal lung auscultation, n (%) | 37 (23.0) | 1 (9.1) | 0.19 |
| Abnormal lung auscultation, n (%) | 52 (32.3) | 2 (18.2) | 0.19 |
|  |  |  |  |
| **Blood test** |  |  |  |
| CRP, median (IQR), mg/L | 60.5 (22.6, 121.0) | 123.5 (71.7, 244.5) | 0.012 |
| Lymphocytes, median (IQR), 10e3/ml | 1100.0 (800.0, 1500.0) | 950.0 (300.0, 1100.0) | 0.036 |
| D-Dimer, median (IQR), μg/L | 424.5 (287.5, 901.5) | 2326.0 (440.0, 5300.0) | 0.092 |
| Fibrinogen ≥ 500, n (%), mg/dL | 119 (73.9) | 8 (72.7) | 0.26 |
| Ferritin, median (IQR), μg/L | 443.5 (201.5, 1132.5) | 2234.0 (113.0, 4355.0) | 0.84 |
|  |  |  |  |
| **RT-PCR SARS-CoV-2** |  |  |  |
| Negative, n (%) | 36 (22.4) | 0 (0.0) | 0.13 |
| Positive, n (%) | 113 (70.2) | 9 (81.9) |  |
|  |  |  |  |
| **Pneumonia features** |  |  |  |
| Pneumonia onset, mean (SD), days | 7.9 (4.1) | 6.8 (4.5) | 0.39 |
| Unilateral, n (%) | 45 (28.0) | 1 (9.1) | 0.17 |
| Bilateral, n (%) | 116 (72.0) | 10 (90.9) |  |
|  |  |  |  |
| **Complications** |  |  |  |
| Hospital admission, n (%) | 129 (80.1) | 11 (100.0) | <0.001 |
| Pulmonary thromboembolism, n (%) | 6 (3.7) | 1 (9.1) | 0.38 |
| Death, n (%) | 0 (0.0) | 11 (100.0) | <0.001 |
|  |  |  |  |
| **Footnotes** n: number, SD: standard deviation, BMI: body mass index, COPD: chronic obstructive pulmonary disease, IQR: interquartile range, CRP: C-reactive protein, RT-PCR: reverse transcription, bmp: beats per minute, rpm: respiratory rate per minute | | | |
|  |  |  |  |
|  |  |  |  |
